# Oxidative Stress and Inflammation in Hepatic Diseases: Current and Future Therapy

**DOI:** 10.1155/2017/3140673

**Published:** 2017-01-22

**Authors:** Karina Reyes-Gordillo, Ruchi Shah, Pablo Muriel

**Affiliations:** ^1^Lipid Research Laboratory, VA Medical Center, 50 Irving Street NW, Washington, DC 20422, USA; ^2^Department of Biochemistry and Molecular Medicine, The George Washington University, 2300 I Street NW, Suite 530, Washington, DC 20037, USA; ^3^Laboratory of Experimental Hepatology, Department of Pharmacology, Cinvestav-IPN, Av. Instituto Politécnico Nacional 2508, Col. San Pedro Zacatenco, 07360, Apartado Postal 14-740, 07000 México, DF, Mexico

Liver disease is a highly prevalent disease that is one of the leading causes of death worldwide. The continuous exposure of the liver to some factors such as viruses, alcohol, fat, and biotransformed metabolites can cause hepatic injury, which can lead to inflammation and liver degeneration. When the injury is sustained for long time, it can cause chronic liver diseases (CLDs), which include a spectrum of disease states ranging from simple steatosis and steatohepatitis (steatosis with inflammation and hepatocyte injury and death) to fibrosis, cirrhosis, and hepatocellular carcinoma (HCC). Multiple evidences indicate that oxidative stress and inflammation are the most important pathogenic events in liver diseases regardless of etiology. Oxidative stress and inflammation are not always harmful; they help phagocytes to kill microorganisms and modulate signaling events through redox regulation. However, unregulated and prolonged imbalance in the liver between the production of free radicals and/or reactive oxygen species (ROS) and their elimination by protective mechanisms (antioxidants) leads to damage of important biomolecules and cells, with potential impact on the whole organism causing many chronic diseases. During liver damage, ROS can induce the generation of proinflammatory genes. A critical component of inflammation is the infiltration of inflammatory cells, like neutrophils, monocytes, and lymphocytes, to the site of stimulus. At the site of inflammation, the activated inflammatory cells release chemical mediators (eicosanoids, cytokines, chemokines, nitric oxide, etc.) that induce tissue damage and augmented oxidative stress and reactive species (superoxide, hydrogen peroxide, hydroxyl radical, etc.). Thus, overexpression of the proinflammatory genes provokes an intracellular signaling cascade that produces more ROS, resulting in a vicious cycle, where increased oxidative stress and inflammatory lesion promote the pathogenesis of liver diseases. A better understanding of the basic pathophysiology underlying the development of steatosis, steatohepatitis, fibrosis, cirrhosis, and HCC is needed, so that better treatments can evolve for liver diseases. Thus, this special issue is dedicated to study the implications of the central roles that oxidative stress and inflammation play in CLDs, as well as the associated current and future therapies.

Antioxidant and anti-inflammatory therapy has been considered to have the possibility of beneficial effects in the management of liver diseases. In this regard, the group of S. Li et al. from China (in “Insights into the Role and Interdependence of Oxidative Stress and Inflammation in Liver Diseases”) summarize the following: (i) the crucial roles of oxidative stress and inflammation in the development of liver damage and (ii) the relationship and interdependence of these processes and also describe (iii) the different herbal medicines or derived compounds targeting oxidative stress and inflammation in various liver diseases. Also from China, the group of Z. Wang et al. (in “Oxidative Stress and Liver Cancer: Etiology and Therapeutic Targets”) provided a review about the development of liver cancer from the perspective of cellular and molecular mechanisms and reported the therapeutic targets of hepatocarcinoma, suggesting that antioxidants are urgently needed to prevent carcinogenesis in the liver. On the other hand, U. S. U. Kumar et al. from Malaysia (in “Redox Control of Antioxidant and Antihepatotoxic Activities of* Cassia surattensis *Seed Extract against Paracetamol Intoxication in Mice: In Vitro and In Vivo Studies of Herbal Green Antioxidant”) reported the protective effect of* Cassia surattensis *seed extract against paracetamol-induced liver toxicity in mice and described the antagonist effects of antioxidants during mild colitis. Moreover, the group of R. Chaphalkar et al. from India (in “Antioxidants of* Phyllanthus emblica* L. Bark Extract Provide Hepatoprotection against Ethanol-Induced Hepatic Damage: A Comparison with Silymarin”) observed that PEE possesses potent antioxidant activity against free radicals and provides significant protection against alcohol-induced liver damage, thus supporting the therapeutic claims made in* Ayurveda* about* Phyllanthus emblica* for treatment of hepatic disorders. In recent times, a new puzzle in medical science has appeared: antioxidants may exert either beneficial or harmful effects depending on the cellular requirement for ROS at a particular situation. In this regard, the group of F. A. Moura et al. from Brazil (in “Colonic and Hepatic Modulation by Lipoic Acid and/or N-Acetylcysteine Supplementation in Mild Ulcerative Colitis Induced by Dextran Sodium Sulfate in Rats”) observed that* N*-acetylcysteine is a promising antioxidant toward alleviating ulcerative colitis and hepatotoxicity, but the combination of lipoic acid and* N*-acetylcysteine in contrast causes hepatic injury and colonic inflammation.

Antioxidants and anti-inflammatories also play a crucial role in metabolic liver diseases. From Brazil, A. Paiva et al. (in “Apolipoprotein CIII Overexpression Induced Hypertriglyceridemia Increases Nonalcoholic Fatty Liver Disease in Association with Inflammation and Cell Death”) demonstrated that persistent hypertriglyceridemia might be more relevant to liver inflammation than intracellular lipid accumulation and that overexpression of apo-CIII increases severity of diet-induced fatty liver disease. This study will be useful to develop new targets to treat metabolic liver diseases. In addition, P. K. Leong and K. M. Ko from China (in “Schisandrin B: A Double-Edged Sword in Nonalcoholic Fatty Liver Disease”) suggest that Schisandrin B, a traditional Chinese herb, may offer potential as a therapeutic agent for NAFLD, due to its antihyperlipidemic, antioxidant, anti-ER stress, anti-inflammatory, and anticarcinogenic activities in cultured hepatocytes in vitro and in rodent livers in vivo.

The reduction of oxidative stress is suggested to be one of the main mechanisms to explain the benefits of subnormothermic perfusion against ischemic liver damage. In this regard, T. Carbonell et al. from Spain (in “Subnormothermic Perfusion in the Isolated Rat Liver Preserves the Antioxidant Glutathione and Enhances the Function of the Ubiquitin Proteasome System”) found that subnormothermic perfusion in the liver can induce oxidative stress concomitantly with antioxidant glutathione preservation, triggering antioxidant mechanisms, protecting against ischemic, hypoxic, and toxic damage. In addition, the group of Y. Zhang et al. from China (in “Hyperglycemia Aggravates Hepatic Ischemia Reperfusion Injury by Inducing Chronic Oxidative Stress and Inflammation”) suggested that chronic oxidative stress, inflammation, and potential malfunction of antioxidative system are the reasons why hyperglycemia aggravates hepatic ischemia reperfusion injury. Biomarkers are necessary for the evaluation of the severity of oxidative stress in I/R injury; thus, the group of H. Li et al. form China (in “Renalase as a Novel Biomarker for Evaluating the Severity of Hepatic Ischemia-Reperfusion Injury”) demonstrated that renalase, a ubiquitous flavin adenine dinucleotide-containing amino oxidase, is a sensitive ROS-responsive gene in hepatocytes which can serve as an efficient and sensitive biomarker for the early warning or evaluation of the severity of hepatic I/R injury.

Liver diseases remain a significant and major health problem around the world. Current therapies in chronic liver diseases are limited and liver transplantation is the only available treatment for end-stage liver disease. This special issue believes to provide novel, effective, and safe approaches to create future antioxidant and anti-inflammatory therapies for patients with CLDs.

*Karina Reyes-Gordillo* *Ruchi Shah* *Pablo Muriel*

